# Identification and Evaluation of *Cryptosporidium* Species from New York City Cases of Cryptosporidiosis (2015 to 2018): a Watershed Perspective

**DOI:** 10.1128/spectrum.03921-22

**Published:** 2023-01-23

**Authors:** Kerri A. Alderisio, Kimberly Mergen, Heather Moessner, Susan Madison-Antenucci

**Affiliations:** a New York City Department of Environmental Protection, Bureau of Water Supply, Water Quality and Innovation Directorate, Watershed Science and Planning, Valhalla, New York, USA; b Parasitology Laboratory, New York State Department of Health, Albany, New York, USA; c Department of Environmental Health Sciences, State University of New York at Albany School of Public Health, Rensselaer, New York, USA; d Rabies Laboratory, New York State Department of Health, Albany, New York, USA; University of Huddersfield

**Keywords:** Cryptosporidium, New York City, cryptosporidiosis, watershed

## Abstract

Watersheds that supply residents with drinking water have the potential for contamination with *Cryptosporidium* oocysts. To evaluate any potential similarities between *Cryptosporidium* species previously found in the New York City (NYC) watershed and those causing disease in NYC, the species were identified in stool specimens from residents with cryptosporidiosis. Genetic analysis was performed on 628 positive stool samples collected from NYC residents between 2015 and 2018 to determine the species present. A total of 547 samples yielded positive results by real-time PCR. Of these samples, 512 (93.6%) were identified to the species level, with 94.7% positive for either Cryptosporidium hominis or Cryptosporidium parvum (56.4% and 38.5%, respectively), including one coinfection. Less common *Cryptosporidium* species identified included C. felis, *C. canis*, *C. ubiquitum*, *C. meleagridis*, and a *Cryptosporidium* sp. chipmunk genotype. Results were evaluated and compared to species and genotypes of *Cryptosporidium* previously identified from stormwater collected within the NYC watershed. While there was overlap with some of the rare species found in case specimens, the prevalence and distribution of species did not suggest a connection between sources previously identified in the watershed and the species causing human cases of cryptosporidiosis in NYC residents.

**IMPORTANCE** It is important to identify the species causing human cryptosporidiosis in a population in order to investigate possible sources or routes of contamination. Many species of *Cryptosporidium* are host-adapted and therefore have the potential to be tracked back to specific sources that can subsequently be managed. There has been no evidence to suggest that the water supply has ever been a source of cryptosporidiosis cases in NYC, and since 2013, the New York City Department of Environmental Protection has further reduced the risk of disease through the use of ultraviolet treatment to inactivate any *Cryptosporidium* present in the source water. However, as one of the largest unfiltered water supplies in the country, it is important to evaluate watershed sources for potential impacts to public health. In this unique study, species of *Cryptosporidium* causing disease in NYC residents were identified and compared with previously identified species from the watershed.

## INTRODUCTION

Cryptosporidiosis is a worldwide concern, as it affects millions of people each year and is a leading cause of diarrhea in humans (1). People can become infected with this waterborne parasite by consuming contaminated food or water, by contact with contaminated water in swimming pools, waterparks, or similar venues, or by contact with infected humans or animals. Cryptosporidium hominis and Cryptosporidium parvum are predominantly responsible for infecting humans (2–4), but other species have also been identified in human specimens (1, 2).

While the watershed that supplies New York City (NYC) residents with drinking water is a potential source of *Cryptosporidium* oocysts, the greatest risk of transport is after heavy rains and snow melt, which can carry animal waste to the waterways through runoff. Additionally, if there is a failure of infrastructure that conveys human waste within the watershed, there is potential for pollution. The New York City Department of Environmental Protection has previously analyzed oocysts from stormwater (5) in its watershed, predominantly at Kensico Reservoir, the terminal, untreated source water for approximately 90% of New York City’s water supply. In addition to identifying *Cryptosporidium* species that cause disease in NYC, this study compared the species identified in patient specimens with oocysts previously detected in the watershed after storms.

## RESULTS

### Demographics and specimens.

During the 4-year study period, a total of 628 stool specimens met the inclusion criteria and were analyzed to identify the species of *Cryptosporidium* present. Of the 628 specimens received from the five boroughs of NYC, a majority were from New York County (274, 43.6%), followed by Kings County (154, 24.5%), Bronx County (117, 18.6%), Queens County (75, 11.9%), and Richmond County (8, 1.3%) (Table 1). There were more males (64%) than females (34.9%) diagnosed with cryptosporidiosis during the 4-year survey. The predominant age range of infected individuals was 26 to 40 years old (37.3%), followed by 41 to 64 years old (24.5%); however, each age bracket was represented.

**TABLE 1:** Demographic distribution of cryptosporidiosis infections in NYC from 2015 to 2018^*a*^

County, *n* (% of total infections)	Age, *n* (% of total)
Bronx, 117 (18.6)	0–10 yrs, 104 (16.6)
Kings, 154 (24.5)	11–25 yrs, 108 (17.2)
New York, 274 (43.6)	26–40 yrs, 234 (37.3)
Queens, 75 (11.9)	41–64 yrs, 154 (24.5)
Richmond, 8 (1.3)	>65 yrs, 26 (4.1)
NA^*b*^	NA, 2 (0.3)

a Of all NYC residents evaluated, 219 (34.9%) were female and 402 (64%) were male; data regarding sex were not available for 7 (1.1%) of residents evaluated.

b NA, information on age, sex, or county of residence not available.

**FIG 1:**
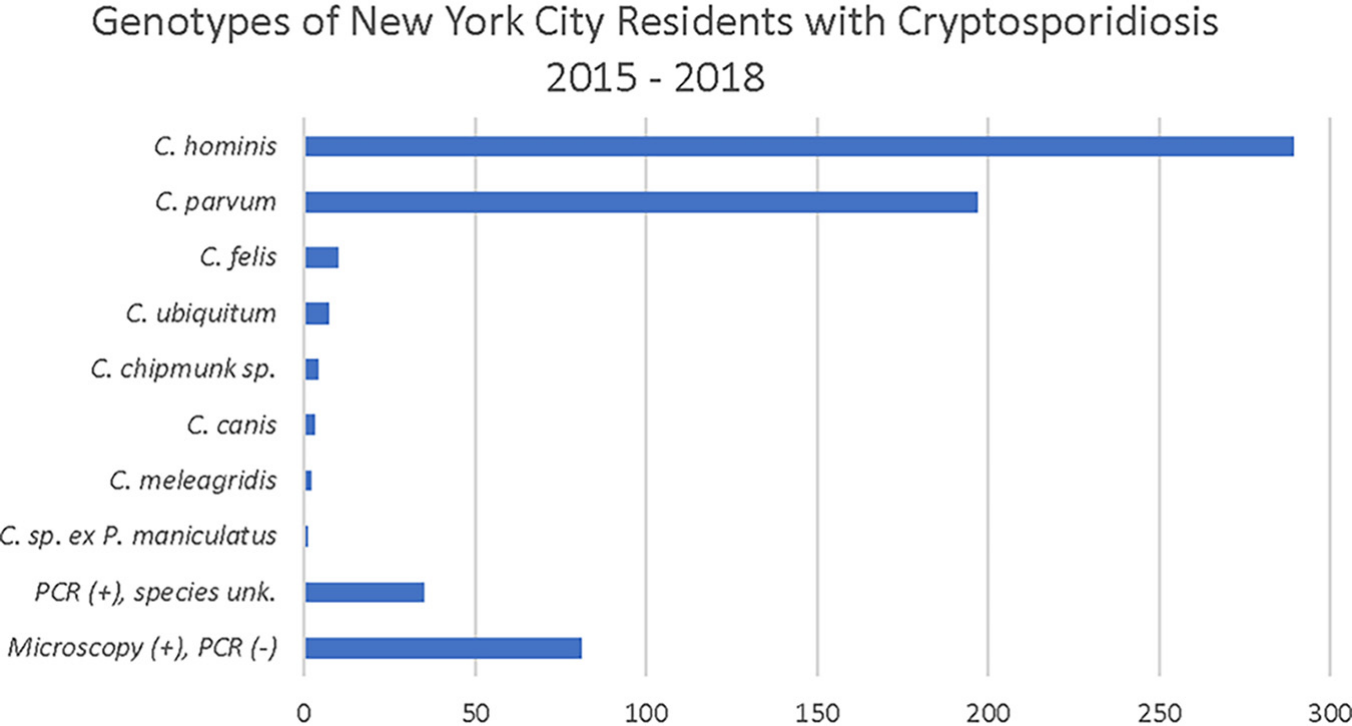
Distribution of *Cryptosporidium* species from 628 stool specimens from NYC residents between 2015 and 2018. Data include one coinfection of C. hominis and C. parvum.

Stool specimens were specifically tested for the presence of C. hominis and C. parvum as well as by a general assay designed to detect *Cryptosporidium* at the genus level (6). Of the 628 specimens tested, 81 were positive by acid-fast staining and/or direct fluorescent antibody (DFA) but did not yield positive results by real-time PCR (RT-PCR) (Fig. 1). It is known that fixatives, particularly formalin, can influence the success of molecular analysis (7–9). In fact, for the samples where molecular methods did not yield a positive result and the fixative was known (*n* =52), 50% were preserved in formalin and 32% in zinc polyvinyl alcohol. Additionally, because molecular methods are known to be more sensitive than microscopy, RT-PCR was performed on stool samples that were both acid fast and DFA negative if the suspected organism indicated by the submitting laboratory was *Cryptosporidium*. Overall, 547 specimens were analyzed by molecular methods. In 2015, the first year of the study, 52 stool specimens received were positive by RT-PCR. In 2016, 2017, and 2018, the number of positive specimens amenable to molecular identification increased to 148, 152, and 195, respectively, coincident with the broad introduction of a new gastrointestinal syndromic panel that included *Cryptosporidium* (10, 11). Consistent with the increase in specimens that could be tested by molecular methods, overall the number of positive *Cryptosporidium* specimens submitted from NYC doubled from 100 in 2015 to 205 in 2016. Submission continued at the higher level in subsequent years, with 182 positive specimens in 2017 and 245 in 2018.

### *Cryptosporidium* sample analysis.

**FIG 2:**
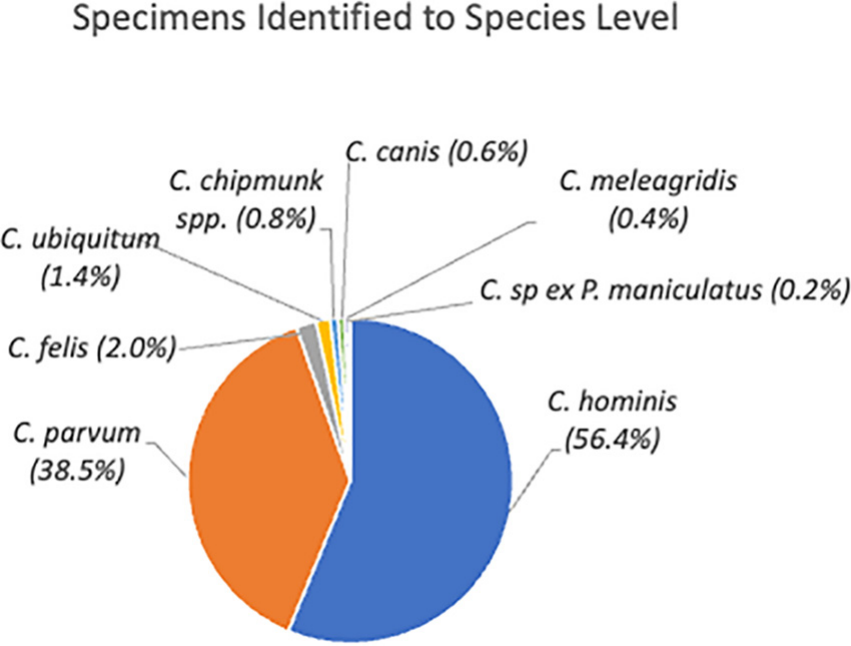
Species identified in 512 cryptosporidiosis specimens from NYC residents from 2015 to 2018.

Of the 547 specimens from NYC residents that were positive for *Cryptosporidium* by RT-PCR, 512 (93.6%) could be identified to the species level (Fig. 2). For the remaining 35 specimens (6.4%) where *Cryptosporidium* was detected in a genus-level RT-PCR assay and both the C. parvum- and C. hominis-specific assays were negative, the species was not identified. C. hominis was detected in 289 (56.4%) of the 512 samples for which a species was identified, and C. parvum was detected in 197 (38.5%) specimens, which included one coinfection in 2017. Overall, C. hominis and/or C. parvum was detected in 88.8% of specimens positive by RT-PCR (with a range of 85.5% to 94.2% per annum) and in 94.7% of specimens for which a species could be positively identified. In total, six other species or genotypes were identified by sequencing of an 800-bp amplification product of the 18S rRNA gene. Species identified included C. felis (10, 2.0%), C. ubiquitum (7, 1.4%), *Cryptosporidium* chipmunk genotype (4, 0.8%), C. canis (3, 0.6%), C. meleagridis (2, 0.4%), and *Cryptosporidium* sp. *ex* Peromyscus maniculatus (1, 0.2%) (Fig. 2). Of these infrequently identified species, only C. felis and *C. ubiquitum* were present in all 4 years tested.

**FIG 3:**
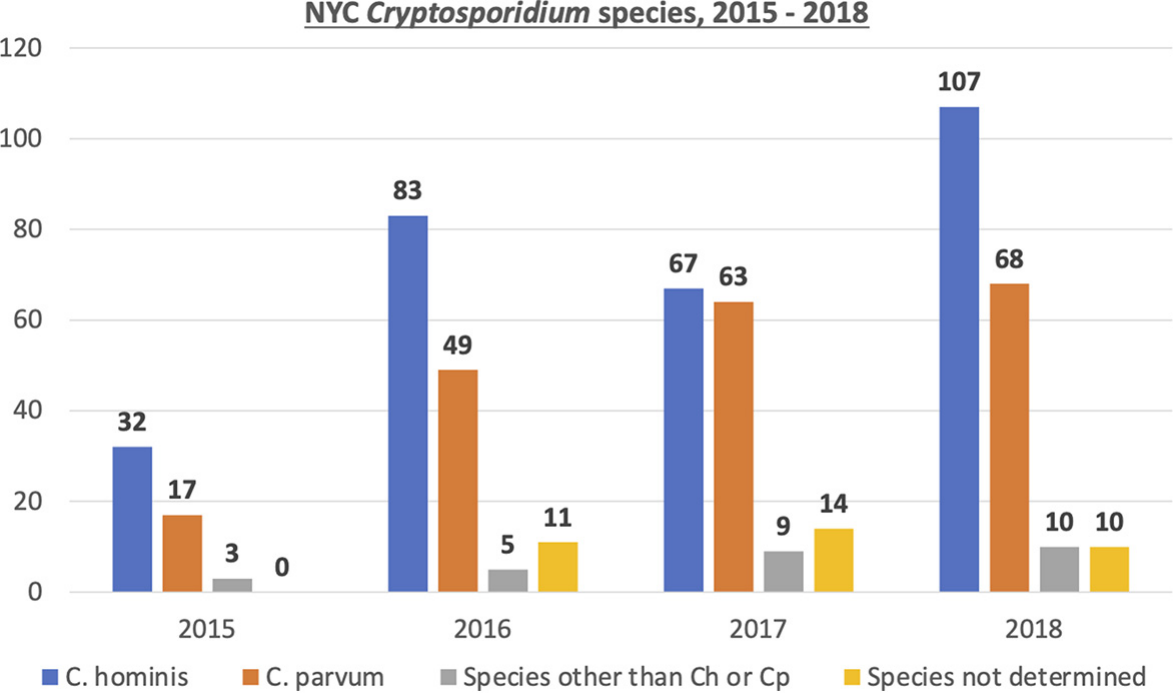
Number of specimens positive by molecular methods annually and the species identified. Sequences could not be obtained for specimens designated *Cryptosporidium* spp., which were positive for *Cryptosporidium* but negative in both the C. hominis- and C. parvum-specific tests.

In 2015, *Cryptosporidium* was identified to the species level in 52 samples. The majority were C. hominis (32, 61.5%) or C. parvum (17, 32.7%) (Fig. 3). The remaining species were C. felis (2) and *C. ubiquitum* (1) (Table 2). For 2016, *Cryptosporidium* was detected by RT-PCR in 148 specimens, and for 137 the species was identified. Again, the majority contained C. hominis (83, 56.1%) or C. parvum (49, 33.1%). Two samples contained *C. ubiquitum*, while C. felis, *C. meleagridis*, and *Cryptosporidium* chipmunk genotype were detected in one specimen each.

**TABLE 2:** Species other than C. parvum and C. hominis detected in stool specimens from NYC residents with cryptosporidiosis

Species	2015	2016	2017	2018	Total
C. felis	2	1	3	4	10
*C. ubiquitum*	1	2	1	3	7
*Cryptosporidium* sp. chipmunk genotype	0	1	1	2	4
*C. canis*	0	0	2	1	3
*C. meleagridis*	0	1	1	0	2
*Cryptosporidium* sp. *ex P. maniculatus*	0	0	1	0	1

In 2017 there was a more equal distribution of C. hominis and C. parvum, with 67 (43.8%) and 63 (41.2%), respectively. For 14 specimens a species could not be identified. Interestingly, there were 139 species identified from 138 specimens, due to one specimen that contained both C. parvum and C. hominis DNA. Additional species identified were the most diverse of the 4 years studied, revealing six different species or genotypes. Among those were three C. felis, two *C. canis*, and one each of *C. ubiquitum*, *Cryptosporidium* chipmunk genotype, *C. meleagridis*, and *Cryptosporidium* sp. ex. *P. maniculatus* (Table 2), which had not been identified in other years. In 2018, of 195 specimens in which *Cryptosporidium* was detected by RT-PCR, 185 were able to be identified to the species level. This produced a distribution similar to 2015 and 2016, where C. hominis was the more predominant species (107, 54.9%) followed by C. parvum (68, 34.9%). Ten specimens (5.1%) contained rare species seen in previous years, including four C. felis, three *C. ubiquitum*, two *Cryptosporidium* chipmunk genotype, and one *C. canis* (Table 2).

## DISCUSSION

Molecular analysis of stool specimens from NYC residents with cryptosporidiosis revealed findings similar to those from other studies in that the dominant species identified were C. hominis and C. parvum (4, 12, 13). Six additional species were identified from NYC resident infections; however, prevalence rates of these other species were much lower.

As the NYC watershed actively supplies millions of residents with drinking water, it is of great importance that the distributed water be clean and not contain infectious pathogens. While there has been, and continues to be, no indication that the water supply has been connected to any cryptosporidiosis cases in NYC, this study set out to identify the species of *Cryptosporidium* associated with stool specimens from NYC residents and compare them with those previously identified from stream stormwater in the watershed prior to treatment. Analysis comparing *Cryptosporidium* species detected in stool specimens with *Cryptosporidium* in partially treated water prior to secondary disinfection was not performed since oocyst detection in the water at this location is rare, and no samples containing oocysts have been positive for *Cryptosporidium* DNA. The Hillview Reservoir outflow (prefinished water entering the distribution system after ultraviolet [UV] disinfection) has been monitored weekly for *Cryptosporidium* oocysts since 2011. Detection of oocysts in this water is rare (4.9%, *n* = 533) and when detected there is usually only a single oocyst. All positive prefinished water specimens were submitted for genotyping analysis; however, all tests have been negative for *Cryptosporidium* DNA using PCR, and thus no genetic material has been available to determine species. Possible explanations for the negative PCR results include oocysts at a level below the detection limit of the PCR assay and the destruction of oocyst DNA after passing through the UV treatment plant. This can lead to a microscopy-positive sample with U.S. EPA Method 1623.1 but a negative PCR result if an oocyst contains no, or damaged, DNA. In addition, the method used for recovering oocysts from slides (14) is not 100% successful, especially if there is only one oocyst on the slide.

This multiyear analysis of NYC residents who were diagnosed with cryptosporidiosis is of public health importance because knowing the most common species associated with human infection can identify the source of the oocysts and inform the management of the source(s) to decrease infection. Here, we analyzed stool specimens from patients with cryptosporidiosis over a 4-year period. Most human infections in this study where species were identified (94.7%) were associated with C. hominis (anthroponotic) and C. parvum (mainly ruminants and humans), and in 88.8% of all RT-PCR-positive specimens, these two species are well documented as the two most significant species infecting humans. Conversely, the predominant species found in studies of NYC stream stormwater within the watershed between 1999 and 2004 were species associated with wildlife (5). Investigators reported the *Cryptosporidium* cervine type (since named *C. ubiquitum*) as most common in stormwater. *C. ubiquitum* is found mostly in scat samples from deer but has also been identified in other hosts (15, 16). While this species is infectious to humans, it is not considered a major human pathogen, as supported by this study of human infections, where *C. ubiquitum* was rarely identified (1.4%). The very infrequent detection of *C. ubiquitum* as a cause of cryptosporidiosis in this study was disproportionate with watershed occurrence, where it was identified much more frequently. This discord supports our conclusion of infection from sources other than those previously identified in the watershed. Other common genotypes of *Cryptosporidium* found in stormwater in the NYC watershed included type W1, later identified from deer mice, a genotype from muskrats, as well as genotypes from skunks, raccoons, and opossums, none of which were found in human infections studied here. As for C. hominis, 6 of the 121 (5%) previously tested stormwater samples were positive (5). However, these samples were all collected from the same stream site over a 2-week period during a series of three storm events. Follow-up testing was performed on a regular basis and all samples were negative. C. parvum was not detected in any of the 121 stormwater samples analyzed.

The comparison of *Cryptosporidium* species and genotypes in this and the stormwater study suggest that even before chlorination and UV disinfection, stormwater samples studied in the streams of the NYC watershed have not resulted in the same types or proportions of *Cryptosporidium* species responsible for 88.8% of RT-PCR-positive cryptosporidiosis infections in NYC (C. parvum and C. hominis). This finding, demonstrating oocyst transmission by means other than drinking water, is supported in the study by Alleyne et al., in which the incidence in NYC was highest among those having person-to-person contact and where 30% of those interviewed (1995 to 2018) reported recent international travel (10). Of the remaining 62 specimens that were *Cryptosporidium* PCR positive, 27 (43.5%) specimens were identified to the species level, with almost half (13) being either C. felis or *C. canis*. Hosts for these *Cryptosporidium* species are predominantly cats and dogs, which are arguably among the most common pet choices for city residents. The remaining 35 specimens were positive for *Cryptosporidium* by microscopy and RT-PCR but negative for C. parvum and C. hominis, and a species could not be determined. While it was not a goal of this study, transmission dynamics could be better understood if genotyping was further investigated. Although epidemiological links were not explored for the patient specimens in this study, future investigations to determine the source of cryptosporidiosis could include both epidemiological evaluation and identification of *Cryptosporidium* subtypes.

Two studies have identified *Cryptosporidium* species in wildlife scat within the NYC watershed, which can potentially be transported from soil to stormwater. Feng et al. in 2007 studied 547 wildlife scat samples from 38 wildlife species (13 of which were rodents) and reported an overall 20.5% positivity rate for *Cryptosporidium* (16). The most common types identified included three rodent genotypes (W1, W3, and deer mouse genotype I) and the cervine genotype (W4), now known as *C. ubiquitum*. Zeigler et al. (17) studied 327 animals of 13 wildlife species (10 of which were rodents) and reported a similar overall *Cryptosporidium* prevalence rate in scat (24%), reinforcing that most wildlife samples are negative for *Cryptosporidium*. The most common types in the Zeigler study were unidentified *Cryptosporidium* spp.; however, these were followed by rodent types and C. parvum. The common finding of rodent types and *C. ubiquitum* in the scat compared to the disproportionately rare, if any, detection of these types in NYC cases continues to suggest sources other than the watershed. Seven of the 77 (9%) samples from the Ziegler study were reported positive for C. parvum, while only 1 of the 111 (0.9%) was positive in the Feng study. This discord may be due to the former scat being collected much further up in the watershed, closer to farms where C. parvum is known to be more prevalent, yet so far from distribution that any impact is unlikely. The single scat detection of C. parvum in the Feng study is consistent with that seen in stormwater, where C. parvum was not detected.

### Strengths.

One of the main strengths of this study is the novel ability to analyze both the potential NYC watershed source data and the NYC resident data for *Cryptosporidium* infections. Comparison of the *Cryptosporidium* species previously found in water sources and the NYC resident samples analyzed here further suggests that infections were not obtained via the NYC watershed but through other modes of contact. Another strength is the large sample size and ability to evaluate infections over a 4-year period. Overall, 94% of PCR-positive samples tested could be identified to the species level.

### Limitations.

Limitations of this study included that not all positive specimens from NYC residents could be analyzed, as there was not enough residual sample to allow testing, or only slides were submitted to the public health laboratory. For those specimens where sufficient volume remained, not all specimens were able to be identified via molecular analysis, which could have impacted the results. In addition, a time period limitation for the comparison of these data (2015 to 2018) to *Cryptosporidium* types in stormwater (5) may exist. It is not known if there was a change in the composition or abundance of wildlife species between the data collection periods for these two studies. If the wildlife population experienced significant temporal variation over these years, then the types of *Cryptosporidium* found in stormwater may have varied as well, potentially impacting conclusions made here.

### Conclusion.

Results from this study indicate two predominant species responsible for cryptosporidiosis in NYC residents: C. hominis and C. parvum. This is consistent with human infectious *Cryptosporidium* species identified elsewhere. However, the species identified as the cause of human infections in NYC are not consistent with, or proportional to, the *Cryptosporidium* species found previously in watershed stormwater or wildlife scat prior to disinfection.

## MATERIALS AND METHODS

The Wadsworth Center Parasitology Laboratory receives specimens for reportable parasitic diseases from hospitals and commercial laboratories that perform diagnostic testing for residents of New York State. When *Cryptosporidium* is identified in a stool specimen, it is submitted to the state lab for confirmation or further identification according to the guidelines in Laboratory Reporting of Communicable Diseases (18).

### Specimens.

Stool specimens submitted to the Wadsworth Center Parasitology Laboratory from 2015 through 2018 to confirm the presence of *Cryptosporidium* were included in the study if the patient’s residence was within the five boroughs of NYC and sufficient residual sample remained after clinical testing. Specimens were excluded if only slides were submitted, as no further testing could be performed to identify the species of *Cryptosporidium* present.

### Stool concentration and microscopy.

Stool samples were concentrated using fecal parasite concentrator (Evergreen Scientific, Buffalo, NY) per the manufacturer’s instructions. Approximately 15 μL of concentrated stool was applied to microscope slides and allowed to dry completely prior to staining by modified acid-fast (Remel, San Diego, CA) and direct fluorescent antibody (DFA; Meridian, Cincinnati, OH). Modified acid-fast stained slides were visualized under bright-field microscopy at 1,000× magnification and were reported positive when size, staining pattern, and morphology were consistent with *Cryptosporidium* oocysts. DFA slides were prepared according to the manufacturer’s instructions and visualized at 200× magnification with a fluorescein isothiocyanate filter. Organisms were reported positive if oocysts stained apple green and were of the correct size and staining pattern.

### Stool preparation and DNA extraction.

Stool specimens were washed with phosphate-buffered saline (PBS), and DNA was extracted as described previously (6). Briefly, 1 mL of stool was washed with PBS, heated for 15 min at 75°C, and homogenized in a bead beater before removing the supernatant for DNA extraction using the NucliSense easyMag automated platform (bioMérieux, Durham, NC). Purified DNA was extracted in a volume of 100 μL.

### PCR amplification and sequencing.

DNA extracted from the stool specimens was tested by a RT-PCR assay that specifically detects C. hominis and C. parvum and also includes degenerate genus-level primers and probe for *Cryptosporidium* (6). To further identify samples that contained species other than C. hominis or C. parvum, nested conventional PCR was performed utilizing the 18S RNA gene as a target. Both the primary and secondary products were amplified using Phire Hot Start II DNA polymerase (Thermo Scientific, Hanover Park, IL) in a 50-μL reaction mixture containing 1 to 10 μL of DNA extracted from a stool specimen. Cycling conditions were as follows: denaturation at 98°C for 30 s followed by 35 rounds of 98°C for 5 s, 55.5°C (primary) or 61.6°C (secondary) for 5 s, 72°C for 15 s, and a final extension at 72°C for 1 min. Products were visualized on a 2% agarose gel stained with ethidium bromide. All products that were of the expected size of 800 bp were purified and sequenced.

PCR products were prepared for sequencing by QIAquick PCR purification (Qiagen, Hilden, Germany) according to the manufacturer’s instructions. Briefly, PCR product of the appropriate size was bound to the QIAquick column, washed with buffer, and then eluted in 40 μL. Concentration of purified DNA was measured using a NanoDrop 1000, and DNA was sequenced at the Wadsworth Center Applied Genomics Technology Cluster using the Sanger dideoxy method. Sequence was analyzed using CLC Main Workbench (Qiagen, Hilden, Germany) and compared to sequences deposited in GenBank. *Cryptosporidium* sequence identification was accepted if there was at least a 98% match between the input sequence and GenBank submissions. This was followed by analysis of the next closest species or genotype, to verify if there was sufficient genetic distance to make a species or genotype determination.

### Data availability.

Nucleotide sequences for species and genotypes were deposited in GenBank under accession numbers OP935190 to OP935212.
